# Radiological and Immunohistochemical Characteristics of PitNETs in 79 Patients Undergoing Neurosurgery

**DOI:** 10.3390/cancers17040666

**Published:** 2025-02-16

**Authors:** Anna Krzentowska, Ryszard Czepko, Dariusz Adamek, Michał Filipowicz, Elżbieta Broniatowska, Filip Gołkowski

**Affiliations:** 1 Department of Endocrinology and Internal Medicine, Medical College, Andrzej Frycz Modrzewski Krakow University, 30-705 Kraków, Poland; fgolkowski@afmmed.edu.pl; 2 Department of Neurosurgery, Medical College, Andrzej Frycz Modrzewski Krakow University, 30-705 Kraków, Poland; rczepko@poczta.onet.pl (R.C.); michal.filipowicz@scanmed.pl (M.F.); 3 Department of Neuropathology, Faculty of Medicine, Jagiellonian University Medical College, 31-008 Kraków, Poland; mnadamek@cyf-kr.edu.pl; 4 Faculty of Health Sciences, Medical College, Andrzej Frycz Modrzewski Krakow University, 30-705 Kraków, Poland; ebroniatowska@uafm.edu.pl

**Keywords:** pituitary neuroendocrine tumors, invasiveness, transcription factors, Knosp scale, Hardy scale

## Abstract

Objective: The objective of the study was to provide radiological and immunohistochemical evaluation of pituitary neuroendocrine tumors (PitNETs), concentrating on their invasiveness, expression of transcription factors, and endocrine function. In addition, it was assessed whether the immunohistochemical type of the tumor had an effect on the invasiveness of the tumor. Methods: We retrospectively analyzed seventy-nine cases of PitNETs. The analysis included their features in magnetic resonance imaging (MRI), invasiveness, and immunophenotype (immunoexpression of transcription factors Pit-1, SF 1, TPit, and the hormones). Results: The most prevalent tumor type in our study was the gonadotroph PitNETs (i.e., tumors derived from the SF 1 lineage), which accounted for over 50.0% of giant tumors. They showed more frequent invasion of the sella turcica (*p* < 0.001) compared with tumors from the Pit-1 and TPit lineages. No statistically significant difference was found in the invasion of the cavernous sinuses by pituitary tumors regardless of the transcription factor from which they originated. Among the potentially aggressive tumors, corticotroph PitNETs dominated, mainly Crooke’s tumor and silent type tumors. Conclusions: PitNETs show invasion toward the cavernous sinuses and sphenoid sinuses to varying degrees. Determining the type of transcription factor is extremely important because it allows us to distinguish between tumors with different biological behaviors and thus allows for the identification of tumors with the potential for aggressive behavior. Tumors that express more than one transcription factor require further investigation.

## Simple Summary

Currently, pituitary adenomas are referred to as neuroendocrine tumors (PitNETs). They are divided on the basis of the transcription factor (TF) from which the tumor originates. Transcription factors from which tumors can originate are: PIT-1, pituitary-specific POU-class homeodomain transcription factor; SF 1, steroidogenic factor 1 and T-PIT, T-box family member TBX19. There may also be tumors without a distinct cell lineage. We radiologically and immunohistochemically evaluated pituitary neuroendocrine tumors. Moreover, we assessed whether the immunohistochemical type of tumor showed any correlation with tumor invasiveness. On the basis of the Knosp scale, the tumor invasion toward the cavernous sinuses was assessed, and on the basis of the Hardy scale, the relation of the pituitary tumor to the sella turcica. In the study group, tumors from the SF 1 factor lineage were the most common and showed invasion toward the sella turcica more often than tumors from other cell lines.

## 1. Introduction

The human pituitary is a gland located within a small bony box, the sella turcica, under the base of the brain. Laterally from the pituitary gland, there are cavernous sinuses. Above the pituitary gland are the optic chiasma, and inferiorly are the sphenoid bone and sphenoidal sinuses. PitNETs (pituitary neuroendocrine tumors, according to the WHO classification of 2022) are tumors of the anterior lobe of the pituitary gland, which account for approximately 16% of all primary brain tumors and for almost 25% of benign primary brain tumors [1]. Tumor diagnosis may be delayed in males, resulting in the tumor achieving a large size before clinical symptoms are apparent [2]. Currently, aggressive pituitary tumors are a big challenge for clinicians. The aggressiveness of a tumor is assessed by its clinical and radiological features and by its behavior during follow-up (the growth rate and response to treatment) [3]. According to the definition, an aggressive pituitary tumor is characterized by its invasiveness (grade 3 or 4 on the Knosp scale), invasion of the sinus of the wedge, extremely rapid tumor growth (growth >20% and at least 2 mm in 6 months), and clinically significant tumor growth despite optimal conventional treatment (growth > 20% despite appropriate surgery, drug treatment, and radiotherapy). Aggressive adenomas are often large tumors, many of which are giant (with the largest diameter ≥ 4 cm) [4]. The WHO has distinguished five subtypes of adenoma, which can take an aggressive course, present with an early recurrence, and be refractory to treatment. These are sparsely granulated somatotropic adenoma, silent corticotropic adenoma, male lactotroph adenoma, PIT-1 positive plurihormonal adenoma, and Crooke’s cell adenoma [5].

Different classifications of pituitary adenomas are used because the management of these tumors requires a multidisciplinary approach (with the team including a pathologist, endocrinologist, neuroradiologist, and neurosurgeon). Pituitary adenomas are therefore classified according to their endocrine function, size, and invasiveness, and the current WHO-recommended classification is based on the transcription factors involved in the development of each tumor type. Hormonally active adenomas are mainly those that produce growth hormone (GH), adrenocorticotropic hormone (ACTH), prolactin (PRL), and rarely thyrotrophic hormone (TSH) [6]. In contrast, tumors producing gonadotropins (FSH, follicle-stimulating hormone; LH, luteinizing hormone) are usually hormonally inactive from a clinical point of view (i.e., they do not present a clinical picture of excessive levels of these hormones), and the main symptoms of these tumors are due to their mass effect and invasive behavior. Based on magnetic resonance imaging data, PitNETs are classified into small (micro < 10 mm), large (macro ≥ 10 mm), and giant (≥40 mm). From a neurosurgical point of view, pituitary tumors are divided into invasive and non-invasive using two scales (i.e., the Knosp scale assessing the penetration of the tumor towards the cavernous sinuses, and the Hardy scale assessing the degree of erosion of the sella turcica and invasion of the sphenoid sinus) [7,8,9].

Due to the important role of transcription factors in the development of these tumors [10], the World Health Organization (WHO) in 2017 proposed the division of PitNETs into Pit-1 lineage tumors (lactotroph, somatotroph, and thyrotroph, plurihormonal Pit-1 positive tumors), TPit lineage tumors (corticotroph), SF 1 lineage tumors (gonadotroph), and tumors without a distinct cell lineage. Thus, PitNETs are classified histopathologically by the WHO according to the hormone content of the tumor cells, which is assessed using immunohistochemical staining [11,12,13]. In 2022, the WHO introduced a modification to the above classification: the category of Pit-1 positive plurihormonal tumor was replaced by two clinically distinct PitNETs: the immature Pit-1 lineage tumor and mature Pit-1 lineage tumor [14]. The most up-to-date version of the WHO classification (5th edition) is accessible as a website beta version dated 2023.

Histopathologically, somatotroph, lactotroph, and corticotroph PitNETs are also divided into sparsely granulated adenomas (SGAs) and densely granulated adenomas (DGAs). This distinction reflects different features of immunopositive hormonal content in adenoma cells and is clinically relevant because sparse granularity adenomas have a more aggressive biological behavior compared with dense granularity adenomas [15]. What is of the highest significance is the clinical behavior of the tumor, and so the prediction of its clinical course is the ultimate goal of any system of classification, both pathological and radiological. In fact, one of the reasons to include the “NET” (neuroendocrine tumors) attribution into the WHO classification of pituitary adenomas is their unpredictable clinical course resulting from their histopathologic features, which is common for all neuroendocrine tumors in any organ (especially the lack of possibility to predict the appearance of metastases, which may happen even in G1, i.e., theoretically “benign” neuroendocrine tumors). As a result, any attempt of “validation”, or rather reassessment, of the particular features of Pit-NETs with regard to their behavior is still one of the most important fields of research on pituitary adenomas (PitNETs).

In the study by Nishioka et al., the role of transcription factors in the accurate diagnosis was evaluated, confirming the need to assess these factors in the precise classification of hormonally inactive tumors as they allow for a more accurate characterization of the biological behavior of pituitary tumors [16]. In the literature, attempts have been made to assess the correlation between the histopathological type of the tumor and its MRI image [17]. The study by Scheithauer et al. evaluated, in one hundred and fifty-three adenomas, the association between tumor type and tumor size, invasiveness, resectability, cell cycle profile, and other factors [18].

In our study, we attempted a retrospective analysis of seventy-nine cases of pituitary tumor. Our study had two main objectives: to radiologically and immunohistochemically (IHC) evaluate pituitary tumors in patients undergoing neurosurgery and to assess whether the immunohistochemical type showed any correlation with tumor invasiveness. For this purpose, the tumor size, tumor volume, its invasiveness, hormonal activity, and transcription factor expression were analyzed.

## 2. Material and Methods

### 2.1. Patients

A retrospective radiological and immunohistochemical analysis of pituitary tumors was performed. The study included a group of seventy-nine patients who underwent surgery at St Raphael’s Hospital in Krakow, Poland, between 2022 and 2024, and who were referred for surgery for a tumor within the sella turcica and in whom a pituitary adenoma was subsequently confirmed by histopathology (HP). Each patient gave their informed consent for the collection of tumor tissue for the study. The patient data were anonymized. The study was approved by the Bioethics Committee of the Andrzej Frycz Modrzewski Krakow University, permission no. KBKA/11/O/2024 on 21 March 2024.

### 2.2. Materials and Methods

#### 2.2.1. Magnetic Resonance Imaging of the Tumor

Each patient was subjected to a pituitary-targeted magnetic resonance imaging (MRI) scan before surgery; in individual cases, a computer tomography (CT) scan of the head was performed due to the fact that MRI was contraindicated. Radiological assessment was based on pituitary MRI with contrast. The thickness of the layers used was 2–3 mm. All standard MRI sequences necessary to diagnose pituitary adenoma were used. Measurements were performed using contrast-enhanced T1-weighted images. A volumetric method was used based on standard software available in the Osirix browser. Based on the MRI image, the tumor was measured in three dimensions (i.e., AP, ML, and CC (cor × sag × cc)), and the tumor volume was calculated. In addition, tumor invasion into the cavernous sinuses was assessed using the Knosp scale, while the invasion toward the sella turcica was assessed according to the Hardy scale. The Knosp classification system is as follows: grade 0—the tumor does not cross the medial line of the internal carotid artery; grade 1—tumor is confined medial to the intercavernous line, crossing the vertical meridian of the carotid siphon in cross-section; grade 2—tumors extend past the intercavernous line but stay within the line tangent to the supracavernous and intracavernous carotid arteries; grade 3—tumors spread lateral to the lateral tangential line; grade 4—tumors totally encase the intracavernous carotid artery. In the Hardy scale, the relation of the pituitary tumor to the sella is defined by grades I to V: intrasellar microadenoma is grade I; macroadenoma causing diffuse enlargement but no perforation of the sellar floor is grade II; those causing focal eruption through the anterior sella surface are grade III; those causing extensive destruction into the sphenoid sinus are grade IV; and those that exhibit CSF (cerebrospinal fluid) or hematogenous spread are grade V. Tumors with suprasellar or parasellar extension were further designated as stages 0 and A to E: stage 0 tumors are intrasellar; stage A tumors reach only the suprasellar cistern; stage B tumors encroach upon the anterior recesses of the third ventricle; stage C tumors elevate the floor of the third ventricle; stage D tumors extend to intradural intracranial growth; and stage E tumors invade the cavernous sinus laterally.

In our study, tumors of Knosp grades 1 and 2 were classified as non-invasive, while grades 3 and 4 tumors were classified as invasive. Analogically, Hardy scale grades 1 and 2 tumors were considered non-invasive, and grades 3 and above were assigned to the invasive group. The patients were referred to a neurosurgeon due to their suffering from symptoms such as headache, dizziness, tinnitus, sudden visual disturbances, and sudden eyelid drooping. A total of 79 consecutive patients underwent transsphenoidal excision of the pituitary tumor via the transnasal approach. All operations were performed by the same neurosurgeon (R.C.) at St Raphael’s Hospital in Krakow.

#### 2.2.2. Immunohistochemical Assessment of the Tumor

The postoperative materials from the resected tumors were examined histopathologically. Immunohistochemical evaluation included the level of pituitary hormones (ACTH, GH, PRL, TSH, LH, FSH) and transcription factors (Pit-1, SF 1, and TPit). Based on the hormones secreted by the adenoma and the clinical picture, the tumors were classified as either hormonally active or inactive. The final histopathological diagnosis followed the guidelines and terminology of the WHO classification (5th edition, website beta version 2022), incorporating the immunoexpression of tropic hormones and the above-mentioned transcription factors.

Sections of 3 um were used. After routine deparaffinization, the rehydration and blocking of endogenous peroxidase activity was conducted with freshly made 3% H_2_O_2_ in methanol for 20 min. At room temperature, sections were microwaved for 30 min using EDTA buffer pH = 9.0 for antigen retrieval and then incubated with the primary antibody—ready to use primary antibody: Anti-ACTH AM487-5M mouse monoclonal (clone: AH26), Anti-Prolactin AM978-5M mouse monoclonal (clone: PRL/2644), Anti-FSH-Beta AM986-5M mouse monoclonal (clone: FSH b/1062), Anti-Thyroid Stimulating Hormone mouse monoclonal AM033-5M (clone: 5404), Anti-Luteinizing Hormone AN787-5M mouse monoclonal (clone: SP132)—for 30 min at room temp. This was followed by Labelled Polymer-HRP anti-mouse (peroxidase labelled polymer conjugated to goat anti-mouse immunoglobulins in Tris-HCl buffer containing stabilizing protein) for 30 min at room temperature, with DAB (diaminobenzidine tetrahydrochloride) as the chromogen applied for 8 min at room temperature. Slides were counterstained with hematoxylin Mayer for 30 s. In the case of GH, PIT1, SF1, and TPIT, citrate buffer pH 6.0 for antigen retrieval was used and then slides were incubated with the Ultra Vision Quanto Detection System V-TL-125-QHD containing 1-blocking serum (Ultra Vision Protein Block) to minimize nonspecific background staining with 5-min incubation. As for the particulars of the antibodies for GH and the transcription factors, the following antibodies were used: Anti-human Growth Hormone AR707-5R polyclonal rabbit—ready to use incubation for 30 min at room temp, Anti-TPit ab243028 mouse monoclonal (clone: CL6251) at a 1:1000 dilution, Anti-Pit-1 ab272639 rabbit polyclonal at a 1:1000 dilution, and Anti-SF-1 antibody ab217317 rabbit monoclonal EPR19744 at a 1:1500 dilution for an incubation of 60 min at room temp. This was followed by the application of the Primary Antibody Amplifier Quanto for 10-min of incubation, and subsequently, the antibody conjugated with HRP-labeled polymer (HRP Polymer Quanto) for 10-min incubation, with DAB (diaminobenzidine tetrahydrochloride) as the chromogen applied for 8 min at room temperature. Slides were counterstained with hematoxylin Mayer for 30 s. To confirm the specificity of the primary antibody, positive and negative control tests were performed, following the manufacturer’s instructions. Sections of human pituitary gland tissue were included as the positive control. The negative control test included substitution of the primary antibody with phosphate buffered saline pH = 7.4.

## 3. Statistics

Continuous variables were described by the mean ± standard deviation (SD), median and interquartile range (IQR), and minimal and maximal values. The Mann–Whitney U-test was used to compare continuous variables between two groups. Categorical variables were presented as frequencies and percentages. The categorical variables were compared using the chi-square test. The *p*-values below 0.05 were considered as statistically significant. In some cases, we applied a power analysis to confirm the statistical results. All of the statistical analyses were performed in R software (version 4.1.0, https://www.R-project.org, accessed on 29 July 2021) and Statistica 13 software (StatSoft Inc., Tulsa, OK, USA).

## 4. Results

There were 32 (40.5%) and 47 (59.5%) female and male patients, respectively, in the group of 79 patients. The mean age ± SD was 57.2 ± 13.9. Two patients were below 30 years of age (2.5%), 10 were 30–40 years old (12.7%), 13 were 41–50 years old (16.5%), 15 were 51–60 years old (19.0%), 23 were 61–70 years old (29.1%), 15 were 71–80 years old (19.0%), and one was over 80 years of age (1.3%). The characteristics of the group are shown in Table 1.

The invasiveness of the tumors was assessed using the Knosp and Hardy scales. Tumors assessed as grades 1 and 2 on the Knosp scale were considered non-invasive, while those graded 3 and 4 on the same scale were considered invasive. Similarly, grades 3 or above 3 tumors classified by the Hardy scale were considered invasive, while those graded 1 and 2 were regarded as non-invasive. Among the invasive tumors, the predominant tumor type was the gonadotroph, as seen when using both the Knosp scale (n = 22) and the Hardy scale (n = 24). A comparison of invasive and non-invasive tumors according to the Knosp scale is presented in Table 2, while Table 3 shows the same comparison according to the Hardy scale.

**Figure 1:**
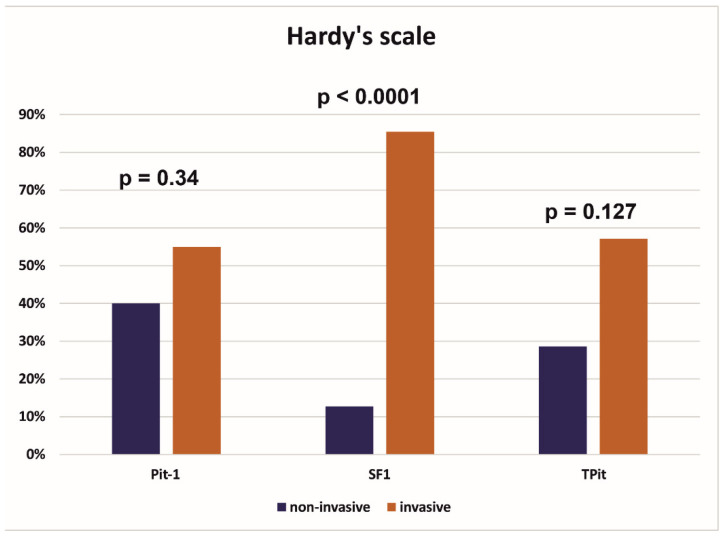
Comparison of invasive and non-invasive tumors in the Hardy scale depending on the transcription factor from which the tumor originated. (Pit-1, pituitary-specific POU-class homeodomain transcription factor; SF 1, steroidogenic factor; TPit, T-box family member TBX19).

Tumors in which a positive expression of individual transcription factors was found were compared in terms of their invasiveness; it was noted that tumors from the SF1 lineage were statistically significantly more frequently invasive than non-invasive, while there were no such differences among tumors from the Pit-1 and TPit lineages (Figure 1).

The patients with tumors were compared by sex and age. The gonadotroph PitNET was more prevalent in males, while the corticotroph PitNET was more common in females, with a statistically significant difference (*p* = 0.035). Among the women, the following observations were made: gonadotroph—n = 14 (43.8%); gonadotroph/lactotroph—n = 2 (6.3%); corticotroph—n = 7 (21.9%); lactotroph—n = 2 (6.3%); null cell adenoma—n = 1 (3.1%); multiple synchronous—n = 1 (3.1%); immature PIT-1—n = 3 (9.4%); mature PIT-1—n = 2 (6.3%). Among the men, the following results were noted: gonadotroph—n = 30 (63.8%); corticotroph—n = 3 (6.4%); lactotroph—n = 2 (4.3%); null cell adenoma—n = 2 (4.3%); multiple synchronous—n = 3 (6.4%); immature PIT-1 positive—n = 4 (8.5%); mature PIT-1 positive—n = 1 (2.1%); somatotroph—n = 1 (2.1%); thyrotroph—n = 1 (2.1%).

There was one case (1.26%) of a microadenoma (<1 cm), seventy-seven cases (97.4%) of macroadenomas, and the data were missing in one case. Giant adenomas (tumors > 40 mm) were present in 11 cases (13.92%). The characteristics of giant tumors are shown in Table 4.

Based on the hormones secreted by the tumor, the endocrine function of the tumors was assessed. The differences between hormonally active and inactive tumors were evaluated in terms of demographic parameters, invasiveness, tumor size and volume, and tumor type (Table 5).

Clinical manifestations in different types of tumors in our group of patients were different depending on hormonal activity. Hormone-inactive tumors (gonadotroph, null sell adenoma, multiple synchronous PitNET, immature Pit-1 lineage tumor, as well as corticotroph tumors of the silent subtype or Crook’s tumor) gave mainly symptoms resulting from the mass effect, i.e., most often visual disturbances, headaches and dizziness. On the other hand, hormonally active tumors gave symptoms dependent on secreted hormones, i.e., lactotroph—mainly menstrual disorders in women; corticotroph—symptoms resulting from hypercorticism; somatotroph—symptoms of acromegaly; mature Pit-1-lineage tumor—mainly symptoms of excess PRL and GH.

On the basis of the histopathological examination, the analysis of the expression of transcription factors was carried out. It was found that some tumors showed a simultaneous expression of several transcription factors (Table 6).

We assessed which tumors originated from the Pit-1 cell line, and the following results were achieved: lactotroph—4 (5.0%); thyrotroph—1 (1.2%); mature Pit-1 lineage tumor—2 (2.5%); immature Pit-1-lineage tumor—7 (8.9%); somatotroph—1 (1.2%). A simultaneous expression geared toward Pit-1 and SF1 was shown by gonadotroph/lactotroph—2 (2.5%). Two patients with a gonadotroph tumor showed a positive expression of the SF1 factor and slight (i.e., at ± expression) in the case of Pit-1.

**Figure 2:**
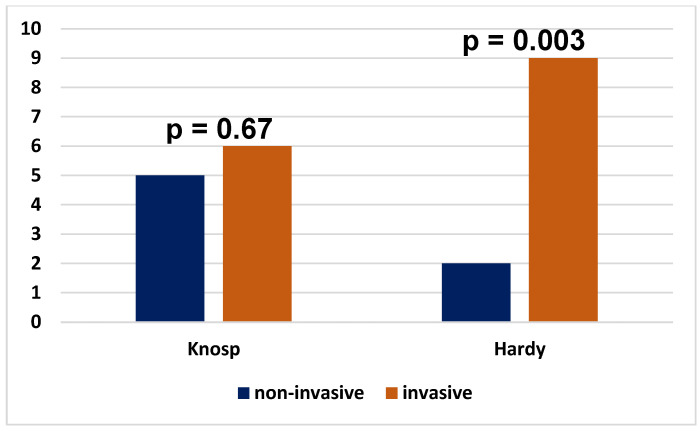
Invasiveness on the Knosp scale and Hardy scale of tumors expressing 2 or 3 transcription factors.

Tumors expressing two and more factors were more often invasive than non-invasive on the Hardy scale, while there was no statistically significant difference between such tumors on the Knosp scale (Figure 2).

Corticotropic PitNETs were then evaluated due to the fact that they may be the cause of Cushing’s disease, but there are also clinically silent tumors with a tendency to aggressive behavior. Among the corticotroph tumors (n = 10) derived from the TPit transcription factor lineage, the following were found: SGCT (sparsely granulated corticotroph tumor)—4 (40.0%); Crooke’s cell tumor—3 (30.0%), silent corticotroph adenomas—2 (20.0%); TPit positive expression but no ACTH expression, missing data—1 (10.0%). The results are presented in Table 7.

Due to the high importance of tumors showing a tendency to aggressive behavior, an analysis of these PitNETs was performed. The following types of adenoma were found, which according to the WHO can have an aggressive course: silent corticotroph adenoma—2; lactotroph adenoma in males—2; PIT-1 positive plurihormonal adenoma—2; Crooke’s cell adenoma—3 (Table 8).

## 5. Discussion

Based on our study, the most common type of PitNET was the gonadotroph tumor (55.69%). Gonadotroph adenomas accounted for 40–60% of clinically non-functioning adenomas [19], and about 20% to 30% of all adenomas. The statement that gonadotroph adenomas are the most frequently detected in patients in the sixth decade of life or older was also confirmed in our study. Similarly, as described in the literature, these tumors were hormonally inactive, and the main symptoms were related to the mass effect [19,20]. In our study, these tumors were more common in males than females, and the mean age at the time of tumor presentation was 60.1 ± 12.8 years. It should be noted that these tumors accounted for one-half of the giant tumors (i.e., reaching more than 40mm), which probably reflected the fact that the delay in the moment of symptom appearance forced the patient to seek medical advice in cases of non-functioning adenomas.

Corticotroph PitNETs are clinically divided into two groups: endocrinologically active tumors presenting with Cushing’s disease or—very rarely—Nelson’s syndrome, and tumors that are clinically non-functioning, the so-called silent corticotroph PitNETs. Corticotropic adenomas showing extensive hyaline changes, the so-called Crooke’s cell adenomas, appear more often to be locally invasive and recurrent [21]. In our study, 10 corticotroph PitNETs were found, including three Crooke’s tumors and two so-called silent tumors. Silent corticotropic PitNETs are characterized by their immunoreactivity for ACTH, although the patients have neither clinical signs of Cushing’s disease nor high levels of ACTH. The majority of such tumors are macroadenomas, and the patients have symptoms of a mass lesion [22,23]. In our study, corticotroph PitNETs accounted for 12.65% of all tumors, the mean age of the patients was 56.7 ± 16.1, and the above lesions were more common in women (70.0%) than in men (30.0%). Similar data were presented by Rak et al. in a study of 2300 cases, where corticotroph PitNETs occurred in more than 70% of women [20].

Lactotroph PitNETs account for approximately 80% of hormonally active tumors and about 40% of all pituitary tumors [6]. In our study, there were four tumors of this type and two tumors secreting PRL and gonadotropins simultaneously. In each case, they were macroadenomas. Of course, most lactotroph PiTNETs are microadenomas but they can also reach large sizes, sometimes even above 40 mm [24,25,26]. Dopamine agonists (DAs) are the first-line treatment, while in the case of drug intolerance or symptoms of pressure on the optic chiasma, surgical treatment is indicated. These are more common in women [24], are microadenomas, and respond to treatment with DAs. In men, on the other hand, tumors reach larger sizes, give symptoms resulting from excess PRL, but also symptoms from the mass effect, hence require surgical treatment. However, it should be emphasized that giant lactotroph PitNETs in men often show an aggressive course and are difficult to treat [26,27,28]. Prolactinomas in males are larger, more invasive, and less sensitive to DAs [29]. In our study, these tumors occurred in young men, and similar results were obtained by other researchers [30]. Giant lactotroph PitNET in men undoubtedly requires additional forms of treatment beyond DAs.

Thyrotroph PitNETs are the least frequent pituitary adenomas. The majority of tumors are invasive macroadenomas [31]. There was one case of a thyrotroph PitNET in our study, which was a macroadenoma in a man, graded as 3 on both the Knosp and Hardy scales.

Somatotroph PitNETs are tumors that secrete growth hormone and give symptoms of acromegaly. In our study, one case of such a tumor was found. However, plurihormonal tumors were also found, which secreted several hormones including growth hormone. Immunohistochemical assessment of pituitary tumors is also necessary in the case of somatotroph PitNET, because some of these tumors (i.e., sparsely granulated somatotropic adenoma) may have aggressive behavior [3,5]. It is therefore important to identify these tumors.

Among the Pit-1 cell line tumors, immature Pit-lineage tumors were the most common. It is noteworthy that some tumors presented more than two transcription factors, and among these were mainly the immature Pit-lineage tumors and gonadotroph tumors.

A plurihormonal Pit-1-positive adenoma is an adenoma that shows immunohistochemical staining for hormones such as GH, PRL, β-TSH, and/or α-SU. These adenomas are usually clinically silent but can sometimes be associated with acromegaly, hyperprolactinemia, or hyperthyroidism. The majority of these adenomas are invasive, aggressive tumors with a high recurrence rate [32]. In our study, plurihormonal Pit-1-positive adenoma tumors mainly secreted PRL, GH, TSH, ACTH, FSH, and LH.

Null cell adenomas are hormonally inactive but give signs of a mass effect. In keeping with the current WHO definition, these adenomas do not show immunoreactivity for any pituitary hormone, nor do they express any of the following transcription factors: Pit-1, SF1, and TPit [33]. Three tumors were found in our study, all of which were macroadenomas; their invasiveness of the Knosp scale was 1, 2, 4 for each tumor, respectively, and on the Hardy scale it was grades 2, 3, 4.

Much research has been devoted to aggressive PitNET behavior [34,35,36]. The search for tumors with the potential for aggressive behavior in our study showed the following results: three Crooke’s cell tumors, two silent corticotroph PitNETs, two lactotroph PitNETs in males, and two plurihormonal Pit-1 positive tumor were found. A number of studies have described the potentially aggressive behavior of Crooke’s cell tumors [37,38]. In our study, Crooke’s tumor was present in two men and one woman. These were macroadenomas, showed suprasellar invasion toward the cavernous and sphenoid sinuses, and the main symptoms of the tumor were visual disturbances and headaches, but also symptoms of Cushing’s syndrome, which has also been described in the literature [37]. A multicenter study of Crooke’s tumor confirmed their poor prognosis and difficulties in normalizing hormones after surgery [39]. Accurate diagnosis of this type of tumor is crucial for postoperative follow-up and treatment planning [40]. Further work is necessary to better understand the pathophysiology of Crooke’s tumors. Among the tumors with the potential to behave aggressively, there are also the so-called silent corticotropic tumors. In our study, two tumors of this type were found. These tumors are usually macroadenomas and are clinically silent, but they give symptoms resulting from the mass effect. Characteristically, these adenomas show a high tendency to apoplexy or hemorrhage [41,42].

An important issue is hormonally inactive tumors. It is important to determine the histopathological type of these tumors because they can have a different clinical course despite the lack of hormonal activity. It should be emphasized that clinically non-functioning pituitary adenomas may be hormonally inactive tumors of differentiated cells, not only gonadotroph adenomas, but also silent corticotroph adenomas and other differentiated silent adenomas [16]. Silent corticotroph adenomas can have an aggressive course. In a study by Nishioka et al. involving five hundred and sixteen hormonally inactive adenomas, about a quarter were silent corticotroph tumors [16]. In our study, there were two cases of such a tumor (i.e., 3.2% of hormonally inactive tumors). Different data may have resulted from our smaller group of patients. Therefore, it is important to determine the transcription factor to confirm cytodifferentiation of these neoplasms. In our study, no differences in size were found between hormonally active and inactive tumors, as some hormonally active tumors such as lactotroph or somatotroph can also reach large sizes.

Giant tumors are also an important issue because they create many problems related to treatment. Unfortunately, complete tumor resection is often not possible. In our study, the gonadotroph PitNETs dominated among giant tumors (6 cases), in addition were two corticotroph tumors, two Pit-1 positive immature tumors, and one multiple synchronous tumor. Similar descriptions have been reported in the literature [43].

The results on the invasiveness of tumors need to be discussed separately. In our group of patients, it was shown that tumors derived from the SF1 factor line were statistically significantly more likely to show a higher severity of invasiveness on the Hardy scale (i.e., a greater tendency toward erosion of the sella turcica), while no such differences were found in the case of tumors from the Pit-1 and TPit lineages. This would explain the main symptoms in patients with tumors derived from the SF 1 factor (i.e., gonadotroph PitNET) such as visual disturbances or headaches. Nevertheless, it should be noted that tumors from the SF-1 lineage are a homogeneous group, while tumors derived from the Pit-1 or TPit lineages are more heterogeneous, which may explain why the latter showed invasion both toward the sella turcica and laterally toward the cavernous sinuses. No statistically significant differences were found in terms of invasiveness toward the cavernous sinuses, regardless of which transcription factor the tumor originated from. However, it should be emphasized that in our study, the results could also have been influenced by the different number of tumors from individual transcription factors.

Our study did not show the effect of gender on the invasiveness of tumors, but this may be due to the small size of the group. Undoubtedly, further research is necessary in this regard. Although there are studies concerning gender differences in the biology of the pituitary gland and in the presentation as well as biology of PA, gender differences regarding the outcome of patients who underwent transsphenoidal resection of PA are poorly understood [2,44,45,46]. In a meta-analysis conducted by Theiler et al. including 40 studies involving a total of 4989 patients, the effect of gender on the results of postoperative control, rate of complete tumor resection, and presence of diabetes insipidus were evaluated [47]. Although individual studies reported data suggesting a gender effect on treatment outcomes, the overall meta-analysis did not confirm these results, and did not show a statistically significant effect of gender.

Ultimately, it should be emphasized that transcription factors play an important role in the prognosis of the type of PitNET, its biology, and subsequent treatment options. Transcription factors also play an important role in the development of other cancers within the brain. Greene et al. studied the role of transcription factors such as ATF5, CEBPB, and CEBPD in the development of brain tumors and other cancers [48]. In turn, Giannopoulou et al. described a selection of oncogenic (GLI-1/2/3, E2F1-8, STAT3, and HIF-1/2) and tumor suppressor (NFI-A/B, TBXT, MYT1, and MYT1L) TFs that are deregulated in gliomas and are subsequently associated with tumor development, progression, and migratory potential [49]. Similarly, the role of factors in the development of glioblastoma has also been studied [50].

It is important to note that one of the most serious limitations of our study was the lack of hormonal testing prior to surgery in all patients. For this reason, they were not included in the analysis. Additionally, the MRI studies before hospital admission were conducted by various diagnostic imaging facilities. Another limitation of our study was the absence of the evaluation of Ki-67 and the p53 protein in some patients, which precluded their inclusion in the comparative analysis. These factors have an impact on the invasiveness of pituitary tumors, which has been described in the literature [51,52,53,54]. These were not included in the study due to the lack of results in all patients, which could have affected the final results. However, it should be emphasized that the histopathological examination was very thoroughly analyzed, on the basis of which the final type of tumor was determined. Despite these limitations, our study provides valuable insights into the prevalence of PitNETs, their hormonal function, and the risk of invasiveness.

Ultimately, it should be emphasized that the assessment of transcription factors is undoubtedly necessary because pathological diagnosis can help the clinician establish postoperative treatment, and above all, early diagnosis and the treatment of potentially aggressive tumors, which has also been highlighted by other authors [12].

## 6. Conclusions

PitNETs continue to represent a significant challenge for clinicians. PitNETs show invasion toward the cavernous sinuses and sphenoid sinuses to varying degrees. Determining the type of transcription factor is extremely important because it allows us to distinguish between tumors with different biological behaviors and thus allows for the identification of tumors with the potential for aggressive behavior. This is particularly important in the assessment of hormonally inactive tumors because there may be potentially aggressive tumors among them, even though they secrete no hormones. Tumors that express more than one transcription factor require further investigation.

## Figures and Tables

**Table 1:** Demographic, radiological, and immunohistochemical characteristics of the study group.

	Overall (N = 79)
**Age**	
Mean (SD)	57.2 (13.9)
Median [Q1–Q3]	60.0 [47.5–68.5]
Min–Max	23.0–82.0
**Gender**	
F	32 (40.5%)
M	47 (59.5%)
**Tumor size AP (mm)**	
Mean (SD)	21.2 (8.40)
Median [Q1–Q3]	20.0 [16.0–25.8]
Min–Max	4.50–50.0
Missing	1 (1.3%)
**Tumor size ML (mm)**	
Mean (SD)	25.5 (8.06)
Median [Q1–Q3]	25.0 [20.0–30.0]
Min–Max	5.50–45.0
Missing	1 (1.3%)
**Tumor size CC (mm)**	
Mean (SD)	24.3 (10.7)
Median [Q1–Q3]	22.0 [17.0–30.8]
Min–Max	4.50–56.0
Missing	1 (1.3%)
**Volume of tumor (cm^3^)**	
Mean (SD)	8.51 (8.66)
Median [Q1–Q3]	5.20 [3.15–10.1]
Min–Max	0.200–50.0
Missing	4 (5.1%)
**Transcriptions factors:**	
Pit-1	21 (26.6%)
SF 1	55 (69.6%)
TPit	14 (17.7%)
**Type of PitNET**	
Gonadotroph	44 (55.69%)
Gonadotroph/lactotroph	2 (2.53%)
Corticotroph	10 (12.65%)
Lactotroph	4 (5.06%)
Multiple synchronous	4 (5.06%)
Somatotroph	1 (1.26%)
Thyrotroph	1 (1.26%)
Null cell adenoma	3 (3.79%)
Mature Pit-1 lineage tumor	3 (3.8%)
Immature Pit-1 lineage tumor	7 (8.86%)
**Hormonal activity of PitNET**	
Non-active	62 (78.48%)
Active	17 (21.52%)
**Hardy scale**	
Non-invasive (grades 1, 2)	15 (19.0%)
Invasive (grades 3 and above)	62 (78.5%)
Missing	2 (2.5%)
**Knosp scale**	
Non-invasive (grades 1, 2)	37 (46.8%)
Invasive (grades 3, 4)	40 (50.6%)
Missing	2 (2.5%)

**Table 2:** Comparison of invasive and non-invasive tumors classified using the Knosp scale (non-invasive—37, invasive—40, no data—2).

	Non-Invasive (N = 37)	Invasive (N = 40)	*p*-Value
**Age**			0.87
Mean (SD)	57.4 (14.1)	56.9 (13.9)	
Median [Q1–Q3]	59.0 [48.0−69.0]	61.0 [46.5–5.3]	
Min–Max	31.0–0.0	23.0–0.0	
**Gender**			0.52
F	14 (37.8%)	18 (45.0%)	
M	23 (62.2%)	22 (55.0%)	
**Max size (mm)**			<0.0001 *
Mean (SD)	22.9 (7.32)	32.1 (9.79)	
Median [Q1–Q3]	22.5 [19.0–0.0]	30.5 [23.8–8.5]	
Min–Max	5.50–0.0	18.0–0.0	
**Tumor volume (cm^3^)**			<0.0001 *
Mean (SD)	4.90 (4.57)	11.8 (10.1)	
Median [Q1–Q3]	3.30 [1.83–3.88]	9.00 [4.60–0.2]	
Min–Max	0.200–0.0	1.70–0.0	
Missing	1 (2.7%)	1 (2.5%)	
**Pit-1**			0.65
Negative	27 (73.0%)	31 (77.5%)	
Positive	10 (27.0%)	9 (22.5%)	
**SF 1**			0.64
Negative	12 (32.4%)	11 (27.5%)	
Positive	25 (67.6%)	29 (72.5%)	
**TPit**			0.44
Negative	30 (81.1%)	35 (87.5%)	
Positive	7 (18.9%)	5 (12.5%)	
**Type of PitNET**			0.37
Gonadotroph	20 (54.1%)	24 (60.0%)	
Gonadotroph/lactotroph	0 (0%)	2 (5.0%)	
Corticotroph	4 (10.8%)	5 (12.5%)	
Lactotroph	3 (8.1%)	1 (2.5%)	
Multiple synchronous	1 (2.7%)	3 (7.5%)	
Somatotroph	1 (2.7%)	0 (0%)	
Thyrotroph	0 (0%)	1 (2.5%)	
Null cell adenoma	2 (5.4%)	1 (2.5%)	
Mature Pit-1 lineage tumor	3 (8.1%)	0 (0%)	
Immature Pit-1 lineage tumor	3 (8.1%)	3 (7.5%)	
**Hormonal activity of PitNET**			0.65
Non-active	28 (75.7%)	32 (80.0%)	
Active	9 (24.3%)	8 (20.0%)	
**Invasiveness on** **the Hardy scale**			<0.0001 *
Non-invasive	14 (37.8%)	1 (2.5%)	
Invasive	23 (62.2%)	39 (97.5%)	

* statistical significance.

**Table 3:** Comparison of invasive and non-invasive tumors according to the Hardy scale (non-invasive—15, invasive—62, no data—2).

	Non-Invasive (N = 15)	Invasive (N = 62)	*p*-Value
**Age**			0.64
Mean (SD)	55.4 (14.7)	57.6 (13.8)	
Median [Q1–Q3]	61.0 [42.0–65.5]	59.5 [49.0–68.8]	
Min–Max	31.0–75.0	23.0–82.0	
**Gender**			0.89
F	6 (40.0%)	26 (41.9%)	
M	9 (60.0%)	36 (58.1%)	
**Max size (mm)**			<0.0001 *
Mean (SD)	18.7 (6.18)	29.9 (9.28)	
Median [Q1–Q3]	20.0 [16.5–23.0]	28.3 [23.0–34.0]	
Min–Max	5.50–29.0	16.0–56.0	
**Volume of tumor (cm^3^)**			<0.0001 *
Mean (SD)	2.22 (1.98)	9.96 (8.96)	
Median [Q1–Q3]	1.65 [1.18–2.93]	8.20 [4.00–12.0]	
Min-Max	0.200–8.00	1.30–50.0	
Missing	1 (6.7%)	1 (1.6%)	
**Pit-1**			0.008 *
Negative	7 (46.7%)	51 (82.3%)	
Positive	8 (53.3%)	11 (17.7%)	
**SF 1**			0.055
Negative	8 (53.3%)	15 (24.2%)	
Positive	7 (46.7%)	47 (75.8%)	
**TPit**			0.23
Negative	11 (73.3%)	54 (87.1%)	
Positive	4 (26.7%)	8 (12.9%)	
**Type of PitNET**			0.011 *
Gonadotroph	5 (33.3%)	39 (62.9%)	
Gonadotroph/lactotroph	0 (0%)	2 (3.2%)	
Corticotroph	2 (13.3%)	7 (11.3%)	
Lactotroph	3 (20.0%)	1 (1.6%)	
Multiple synchronous	0 (0%)	4 (6.5%)	
Somatotroph	1 (6.7%)	0 (0%)	
Thyrotroph	0 (0%)	1 (1.6%)	
Null cell adenoma	0 (0%)	3 (4.8%)	
Mature Pit-1 lineage tumor	2 (13.3%)	1 (1.6%)	
Immature Pit-1 lineage tumor	2 (13.3%)	4 (6.5%)	
**Hormonal activity of PitNETs**			0.084
Non—active	9 (60.0%)	51 (82.3%)	
Active	6 (40.0%)	11 (17.7%)	
**Invasiveness on the Knosp scale**			<0.0001 *
Non-invasive	14 (93.3%)	23 (37.1%)	
Invasive	1 (6.7%)	39 (62.9%)	

* statistical significance.

**Table 4:** Radiological and immunohistochemical characteristics of giant tumors (tumor size > 40 mm).

Age	Sex	AP (mm)	ML (mm)	CC (mm)	KS	HS	V cm^3^	Type PitNET	PRL	ACTH	GH	TSH	LH	FSH	Pit-1	SF 1	TPit
62	F	33	27	41	1	4D	18	Gonadotroph	0	0	0	0	0	0	0	++	0
64	M	40	37	45	4	4E	33	Multiple Synchronous	0	0	0	0	+	0	0	+	0
65	M	29	36	46	4	4D	21	Gonadotroph	0	0	0	0	0	0	0	+++	0
73	F	41	41	43	4	4E	25	Gonadotroph	0	0	0	0	0	0	0	+++	0
62	M	33	44	35	4	4A		Immature Pit-1	0	0	0	0	0	0	+	−/+	0
43	M	31	29	51	4	4E	21	Immature pit-1	0	0	0	0	0	0	+	0	0
57	F	30	45	31	3B	4E	21	Corticotroph	0	+	0	0	0	0	0	0	+
63	F	26	34	43	4	4E	19	Corticotroph	0	0	0	0	0	0	0	0	+
44	M	37	44	56	4	4E	50	Gonadotroph	0	0	0	0	0	+	0	+	0
70	F	50	38	43	4	4E	33	Gonadotroph	0	0	0	0	0	+	0	+	0
57	M	32	40	40	4	4D	13	Gonadotroph	0	0	0	0	0	+	0	+	0

Legends: KS, Knosp scale; HS, Hardy scale; GH, growth hormone; ACTH, adrenocorticotropic hormone; PRL, prolactin; TSH, thyrotrophic hormone; FSH, follicle-stimulating hormone; LH, luteinizing hormone; PIT-1, pituitary-specific POU-class homeodomain transcription factor; SF 1, steroidogenic factor 1; T-PIT, T-box family member TBX19.

**Table 5:** Comparison of hormonally active and inactive PitNETs.

	Hormonally Inactive (N = 62)	Hormonally Active (N = 17)	*p*-Value
**Age**			0.089
Mean (SD)	58.7 (13.0)	51.8 (15.9)	
Median [Q1–Q3]	62.0 [50.0–69.0]	52.0 [39.0–64.0]	
Min–Max	27.0–82.0	23.0–77.0	
**Gender**			0.24
F	23 (37.1%)	9 (52.9%)	
M	39 (62.9%)	8 (47.1%)	
**Max size (mm)**			0.37
Mean (SD)	28.3 (9.75)	25.8 (9.80)	
Median [Q1–Q3]	26.0 [22.0–33.0]	23.0 [20.0–29.0]	
Min–Max	5.50–56.0	8.00–45.0	
Missing	1 (1.6%)	0 (0%)	
**Tumor volume (cm^3^)**			0.13
Mean (SD)	8.94 (8.72)	7.05 (8.53)	
Median [Q1–Q3]	5.60 [3.23–10.8]	4.00 [1.40–8.60]	
Min–Max	0.800–50.0	0.200–33.0	
Missing	4 (6.5%)	0 (0%)	
**Type of PitNETs**			<0.0001 *
Gonadotroph	44 (71.0%)	0 (0%)	
Gonadotroph/lactotroph	0 (0%)	2 (11.8%)	
Corticotroph	5 (8.1%)	5 (29.4%)	
Lactotroph	1 (1.6%)	3 (17.6%)	
Null cell adenoma	3 (4.8%)	0 (0%)	
Multiple synchronous	3 (4.8%)	1 (5.9%)	
Thyrotroph	0 (0%)	1 (5.9%)	
Somatotroph	0 (0%)	1 (5.9%)	
Mature Pit-1-lineage tumor	0 (0%)	3 (17.6%)	
Immature Pit-1 lineage tumor	6 (9.7%)	1 (5.9%)	
**Invasiveness on the Hardy scale**			0.084
Non-invasive	9 (14.5%)	6 (35.3%)	
Invasive	51 (82.3%)	11 (64.7%)	
Missing	2 (3.2%)	0 (0%)	
**Invasiveness on the Knosp scale**			0.65
Non-invasive	28 (45.2%)	9 (52.9%)	
Invasive	32 (51.6%)	8 (47.1%)	
Missing	2 (3.2%)	0 (0%)	

* statistical significance.

**Table 6:** Tumors showing simultaneous expression of 2 or 3 transcription factors (n = 11).

Type of Pit-NET	PRL	ACTH	GH	TSH	LH	FSH	Pit-1	SF 1	TPit
Gonadotroph	0	0	0	0	0	0	−/+	++	0
Immature Pit-1 lineage tumor	0	0	0	0	0	0	+	−/+	0
Gonadotroph	0	0	0	0	0	1	−/+	+	0
Immature Pit-1 lineage tumor	0	0	0	0	0	0	+	+	0
Immature Pit-1 lineage tumor	0	0	0	0	0	0	+	−/+	−/+
Gonadotroph	0	0	0	0	0	0	−/+	+	0
Gonadotroph/lactotroph	1	0	0	0	0	1	+	+	0
Gonadotroph/lactotroph	1	0	0	0	1	1	+	+	0
Immature Pit-1 lineage tumor	0	0	0	0	0	0	+	−/+	+
Mature Pit-1 lineage tumor	1	1	1	0	1	1	+	+	+
Immature Pit-1 lineage tumor	0	0	0	0	0	0	−/+	+	0

Legends: GH, growth hormone; ACTH, adrenocorticotropic hormone; PRL, prolactin; TSH, thyrotrophic hormone; FSH, follicle-stimulating hormone; LH, luteinizing hormone; PIT-1, pituitary-specific POU-class homeo-domain transcription factor; SF 1, steroidogenic factor 1; T-PIT, T-box family member TBX19.

**Table 7:** Characteristics of corticotroph PitNETs (tumors with positive expression of TPit transcription factor).

Age	Sex	AP (mm)	ML (mm)	CC (mm)	Knosp Scale	Hardy Scale	V cm^3^	Subtype	ACTH
41	F	14	17	11	2	2A	1.9	SGCT	0
31	M	20	25	20	4	3B	3.7	Crooke	1
72	F	23	22	20	4	3E	4.6	SGCT	0
71	M	No data	No data	No data	No data	No data	No data	Silent	0
38	F	4.5	5.5	4.5	1	1A	0.8	SGCT	1
77	M	34	21	18	1	3C	6.2	Crooke	1
57	F	30	45	31	3B	4E	21	No data	1
63	F	26	34	43	4	4E	19	Silent	0
49	F	27	26	27	2	3C	8.6	Crooke	1
68	F	20	27	21	3A	3	6	SGCT	1

**Table 8:** Radiological and immunohistochemical characteristics of tumors with the potential for aggressive behavior (n = 11).

Age	Sex	AP (mm)	ML (mm)	CC (mm)	Knosp Scale	Hardy Scale	V cm^3^	Type of PitNET	Sub Type	PRL	ACTH	GH	TSH	LH	FSH	Pit-1	SF 1	TPit
35	M	20	22	29	1	2C	8	Lactotroph		1	0	0	0	0	0	+		0
23	M	28	35	29	4	4D	12	Lactotroph		1	0	1	0	0	0	+	0	0
31	M	20	25	20	4	3B	3.7	Corticotroph	CA	1	1	1	0	0	0	0	0	+++
77	M	34	21	18	1	2C	6.2	Corticotroph	CA	0	1	0	0	0	0	0	0	+
49	F	27	26	27	2	3C	8.6	Corticotroph	CA	0	1	0	0	0		0	0	+
63	F	26	34	43	4	4E	19	Corticotroph	Silent	0	0	0	0	0	0	0	0	+
71	M	No date	No date	No date	No date	No date	No date	Corticotroph	Silent	0	0	0	0	0	0	0	0	+++
75	M	6	17	6	1	2A	0.5	Plurihormonal		1	1	1	0	1	1	+	+	+
66	F	8	8	6	0	no date	0.2	Plurihormonal		1	0	1	1	0	0	+	0	0

Legend: CA, Crooke’s cell adenoma; GH, growth hormone; ACTH, adrenocorticotropic hormone; PRL, prolactin; TSH, thyrotrophic hormone; FSH, follicle-stimulating hormone; LH, luteinizing hormone; PIT-1, pituitary-specific POU-class homeo-domain transcription factor; SF 1, steroidogenic factor 1; T-PIT, T-box family member TBX19.

## Data Availability

The datasets used and/or analyzed during the current study are available from the corresponding author upon reasonable request.
